# Safety of Initiating Sodium-Glucose Cotransporter-2 Inhibitors in Patients with Heart Failure or Type 2 Diabetes and a History of Urinary Tract Infections

**DOI:** 10.3390/healthcare14030318

**Published:** 2026-01-27

**Authors:** Jacqueline Rever, Noman Khalid, Caitlin Kulig, Justina Girgis

**Affiliations:** 1 St. Joseph’s University Medical Center, Paterson, NJ 07503, USA; 2 Ernest Mario School of Pharmacy, Rutgers, The State University of New Jersey, Piscataway, NJ 08854, USA

**Keywords:** SGLT2 inhibitors (SGLT2is), urinary tract infections (UTIs), heart failure, type 2 diabetes mellitus (T2DM), HFrEF GDMT, HFpEF

## Abstract

**Background**: Despite being a pillar of heart failure (HF) management, the guideline-directed initiation of sodium-glucose cotransporter-2 inhibitors (SGLT2is) may be challenging due to the barrier of associated urinary tract infections (UTIs). Although there is a known risk, it remains unclear whether UTI incidence differs between patients with and without a prior history of UTIs. **Methods**: This study aimed to evaluate the risk–benefit profile of initiating an SGLT2i in patients with a history of UTIs. This retrospective, single-center healthcare system cohort analysis included adult patients hospitalized and taking an SGLT2i between 1 January 2020, and 31 August 2024. The included patients were divided into two cohorts: patients with and without a history of UTI pre-SGLT2i (described in this study as UTI-naive). Patients with urogenital structural abnormalities, indwelling catheters, or high-risk profiles were excluded. The primary outcome was the incidence of UTIs post-SGLT2i initiation. Secondary outcomes included the number of UTIs within 30, 60, and 90 days after starting an SGLT2i. **Results**: A total of 280 patients were evaluated for this study, of which 250 were included for analysis. Of those, 197 were UTI-naive, and 53 had a history of UTI pre-SGLT2i use. The most utilized SGLT2i was empagliflozin (75.6%). Amongst the cohorts, 20.4% of the UTI-naive patients developed a UTI post-SGLT2i versus 30.2% in patients with a historical UTI (*p* = 0.13). **Conclusions**: There was no significant difference in UTIs developed up to 90 days post-SGLT2i initiation, regardless of previous infections, suggesting that a history of UTI should not be a barrier to differing first-line therapy.

## 1. Introduction

Sodium-glucose cotransporter-2 inhibitors (SGLT2is) have become a pillar in the management of various common disease states. With initial approval for the improvement of glycemic control in type 2 diabetes mellitus (T2DM), the SGLT2i class has since expanded its indications to include heart failure (HF) and chronic kidney disease (CKD).

The American Heart Association (AHA) and American College of Cardiology (ACC) guidelines for the treatment of heart failure recommend SGLT2is as one of the four medication classes included as guideline-directed medical therapy (GDMT) for heart failure with reduced ejection fraction (HFrEF) [1]. Empagliflozin, dapagliflozin, and sotagliflozin have each demonstrated reductions in HF-related hospitalizations and cardiovascular mortality among patients with HFrEF [2,3,4]. These findings have contributed to the Class I, Level A guideline recommendation for initiation of SGLT2 inhibitors in this population [2,3,4]. More recently, studies have looked at the utilization of SGLT2i in heart failure with moderately reduced ejection fraction (HFmrEF) and heart failure with preserved ejection fraction (HFpEF), which also found benefits in cardiovascular mortality and hospitalization in these patients [5,6]. Based on these findings, the heart failure guidelines recommend SGLT2i initiation across the entire ejection fraction spectrum [1,2,3,4,5,6].

GDMT in heart failure is extremely important. In HFrEF, for example, it is estimated that the use of all four pillars of GDMT, which includes SGLT2is, reduces all-cause mortality by 73% versus no treatment [7]. Yet despite the importance of GDMT in HF in preventing death and hospitalizations, data still support that the use of GDMT is wholly underprescribed. Based on claims data, roughly 42% of patients are not prescribed any GDMT within 30 days of HF hospitalizations, and at 1 year, roughly 45% of patients are either no GDMT or GDMT monotherapy [8,9].

During the approval and post-marketing surveillance studies of SGLT2is, one of the major adverse reactions associated with SGLT2i use was urinary tract infections (UTIs) [10]. When initially developed for glycemic control in T2DM, through inhibiting the SGLT2i transporters, these medications were able to reduce the reabsorption of filtered glucose into the serum and increase urinary glucose excretion [10]. Consequently, with more glucose passing through the urinary tract, there is an increased risk for infection as bacteria tend to thrive in high-glucose environments [10]. Specifically looking at the most prescribed SGLT2i, empagliflozin, the incidence of UTIs, including asymptomatic bacteriuria and cystitis, was increased when compared to placebo (empagliflozin 10 mg, 9.3% vs. placebo, 7.6%) [10]. The risk of UTIs was shown to be higher in patients with specific risk factors, such as females or a history of chronic or recurrent urinary tract infections [10].

With known risk factors and evidence showing SGLT2i utilization increases the incidence of UTIs, this has potentially led to some hesitation in prescribing for HF despite the proven mortality and hospital reductions. The studies conducted linking SGLT2i use and UTIs were mainly focused on initial infection and highlighted patients with T2DM [2,3,4,5,6]. To our knowledge, there is little data regarding the incidence of UTIs after starting an SGLT2i in patients with HF and previous UTIs. This study aimed to understand the risk-benefit profile of initiating an SGLT2i in patients with a history of UTIs versus those with no history of UTIs, specifically regarding the incidence of post-SGLT2i infections.

## 2. Materials and Methods

### 2.1. Study Design

This retrospective, single-healthcare-system, cohort study was approved by the investigational review board (IRB) committee, with a waiver of informed consent granted due to the retrospective nature of the study and the analysis of de-identified data. Data were collected through chart reviews on the electronic medical records (EMRs) of eligible patients.

### 2.2. Enrollment

Patients were included in this study if they were at least ≥18 years old, were admitted to the hospital, and were taking an SGLT2i. Patients were excluded for the following reasons: (1) genital structural abnormalities, (2) presence of an indwelling catheter, or (3) classified as high-risk patient populations, such as pregnant women, children, or incarcerated individuals.

A pharmacy-generated medication administration record, including SGLT2is, for patients hospitalized between 1 January 2020 and 31 August 2024, was created to assess for eligible participants for inclusion. The International Classification of Diseases, Tenth Revision (ICD-10) codes for heart failure and urinary tract infections were also utilized for the initial data collection report. The goal sample size was 250 eligible participants. For statistical analysis, the included patients were to be separated into two cohorts depending on their history of UTIs.

### 2.3. Clinical Outcomes

The primary outcome assessed the incidence of UTIs after initiating an SGLT2i. A UTI was stringently defined by the presence of a positive urine culture coupled with a concomitant clinical diagnosis of UTI or documented symptoms necessitating antibiotic treatment. Asymptomatic bacteriuria alone was not considered a UTI event. The secondary outcomes stratified the number of UTIs developed within 30, 60, and 90 days post-SGLT2i initiation. An additional key secondary objective involved the comparison of identified uropathogens in urine cultures preceding SGLT2i initiation with those identified in post-SGLT2i UTI events. Subgroup analyses were performed based on disease state (heart failure, T2DM, and CKD) and each SGLT2i and dose prescribed.

### 2.4. Statistical Analysis

Baseline characteristics of the study population were summarized using descriptive statistics, with continuous variables presented as mean ± standard deviation (SD) and categorical variables as counts and percentages. Continuous data, such as age, body mass index (BMI), and left ventricular ejection fraction (LVEF), were analyzed with an unpaired *t*-test. Finally, the spectrum of identified uropathogens in urine cultures pre-SGLT2i and post-SGLT2i was analyzed. Frequencies and percentages of specific pathogens were compared using chi-square tests. The confidence interval was defined as 95%, and results were considered statistically significant if the *p*-value was less than 0.05.

## 3. Results

### 3.1. Demographic and Baseline Characteristics

**Figure 1:**
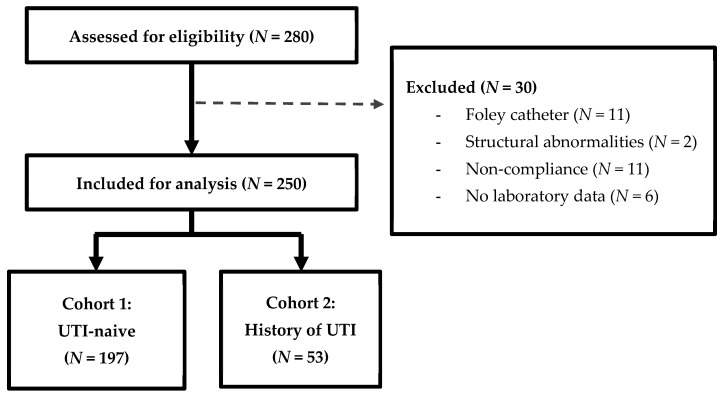
Participant enrollment.

Of the 280 patients evaluated for inclusion, 250 patients were included for analysis (Figure 1). Of those, 197 patients were categorized as UTI-naive, having no historical UTIs prior to SGLT2i, and 53 were classified in the history of UTI cohort. Patient characteristics are summarized in Table 1. The mean age was 67 ± 12.6 years for patients who were UTI-naïve and 65 ± 15.1 years for those with a history of UTIs. There were significantly more males in the cohort with a history of UTIs (84.9% vs. 53.8%). The most commonly prescribed SGLT2i was empagliflozin (75.6%), followed by dapagliflozin (19.2%), ertugliflozin (3.6%), and canagliflozin (0.4%). A majority of patients had diabetes (84.0%), with a significantly higher proportion in patients with a history of UTIs. There were 108 patients (43.2%) with documented heart failure, 52 of whom (48.1%) had HFrEF.

### 3.2. Incidence of UTIs Post-SGLT2i

There was a total of 56 patients with documented UTIs after initiating an SGLT2i, of whom 40 were UTI-naïve and 16 had a historical UTI. There was no statistically significant difference between UTI incidence post-SGLT2i when comparing UTI-naive patients versus patients with a history of UTIs (20.4% vs. 30.2%, *p* = 0.13). Additional analysis is described in Table 2.

### 3.3. Number of UTIs 90 Days Post-SGLT2i

The secondary outcome assessed the timing of UTIs up to 90 days post-SGLT2i. As noted in Table 2, the majority of documented infections occurred more than 90 days after starting an SGLT2i for all patients, UTI-naive, and history of UTI patients (75.8%, 77.3%, and 66.7%, respectively). Notably, there were numerically more UTIs within the first month of SGLT2i usage in patients with a history of UTI; however, the results were not statistically significant.

### 3.4. Patients with Diabetes

Since an SGLT2i is also indicated to treat type 2 diabetes and increases the risk of developing UTIs, the authors wanted to analyze UTI incidence in this population. Of the 210 patients with T2DM, 25.2% developed a UTI (Table 3). The majority, and statistically more, of post-SGLT2i UTIs were in the UTI-naive cohort (71.7%) versus the patients with a history of UTI (28.3%).

### 3.5. Patients with Heart Failure

Among the 250 included patients, 108 had heart failure, 22 of whom (20.3%) developed a UTI after starting an SGLT2i (Table 3). Of note, only 2 participants did not have a comorbidity of type 2 diabetes. There was a statistically higher number of UTIs developing in patients with HF who were UTI-naive (68.2%) versus patients with a historical UTI (31.8%, *p* = 0.04). This significance did not carry over to the 8 patients with HFrEF and documented UTI, of whom 62.5% were UTI-naive, and 37.5% had a historical UTI (*p* = 0.62).

### 3.6. Pathogens Pre-SGLT2i vs. Post-SGLT2i

The most common pathogen detected in urine cultures was Escherichia coli in both pre-SGLT2i (46.9%) and post-SGLT2i (38.0%) (Table 4). There were higher frequencies of less common uropathogens associated with post-SGLT2i usage, such as Candida spp. and Enterococcus faecalis. The direct comparison of UTI pathogens for patients with pre- and post-SGLT2i UTIs can be found in Supplemental Table S1.

## 4. Discussion

This retrospective, single healthcare system, cohort study assessed the safety of initiating an SGLT2i in patients with a history of urinary tract infections and sought to determine whether a history of UTIs should be a reason for withholding SGLT2i therapy in patients. Our findings confirmed that SGLT2i use does increase the incidence of UTIs overall, but there is no statistical difference in the incidence of infection when comparing patients with and without a history of UTIs (30.2% vs. 20.4%, *p* = 0.13). The data did show that patients with a history of a UTI pre-SGLT2i use were more likely to develop infections with more gram-positive and fungal pathogens compared to initial UTIs, which is similar to previous knowledge regarding secondary uropathogens. The one caveat of this study’s findings was that the overall number of UTIs in each group was relatively high (20–30%) compared to previous studies on SGLT2i-associated UTIs, which typically ranged <10% [10]. Based on our data, an SGLT2i can be initiated as part of the first-line management for heart failure regardless of UTI history.

The current literature available shows evidence demonstrating the correlation between SGLT2i use and the development of an initial UTI [11,12,13]. Due to this knowledge, there may be hesitation in prescribing an SGLT2i in patients with historical UTIs. These concerns have potentially also led to fewer studies describing the utilization of an SGLT2i in this patient population due to the possibility of further increasing the risk of secondary UTIs. The potential risk of therapies should always be balanced by the potential benefit.

However, this lack of confidence in prescribing one of the four pillars of HFrEF GDMT may lead to withholding the combined morbidity and mortality benefit with the addition of an SGLT2i. The most recent 2022 AHA/ACC guidelines for the management of HFrEF encourage the use of all four GDMT drug classes due to their combined estimated 73% reduction in all-cause mortality when compared to no treatment [1].

When considering the additional indications for SGLT2i, both the diabetes and chronic kidney disease treatment guidelines emphasize the benefits of SGLT2i use with Class 1A recommendations [14,15]. The 2025 Standards of Care Diabetes guidelines recommend an SGLT2i or glycogen-like peptide 1 receptor agonist (GLP-1 RA) for patients with T2DM and risk factors for or known atherosclerotic cardiovascular disease (ASCVD), regardless of hemoglobin A1c or metformin use [14]. When compared to dipeptidyl peptidase-4 inhibitors (DPP4i), the use of an SGLT2i was associated with significantly lower risk of major adverse cardiac events (MACEs, HR 0.85, 95% CI 0.81–0.90), all-cause mortality (HR 0.79, 95% CI 0.73–0.85), and heart failure hospitalizations (HR 0.74, 95% CI 0.66–0.83) [14,16]. Even compared to GLP-1 RA with cardiovascular benefits, patients receiving an SGLT2i had significantly lower heart failure hospitalization rates (HR 0.79, 95% CI 0.67–0.92) [14,16]. The most recent 2024 Kidney Disease Improving Global Outcomes (KDIGO) guidelines for the management of CKD also include SGLT2i therapy as a first-line recommendation [15]. Several studies, including the EMPA-KIDNEY and DAPA-CKD trials, have shown SGLT2i use can reduce the risk of kidney disease progression, acute kidney injury, and cardiovascular death [17,18]. These benefits were seen regardless of diabetes status, kidney disease etiology, or eGFR levels [17,18]. Based on the proven benefits, KDIGO makes a Class 1A recommendation for starting an SGLT2i in patients with an eGFR ≥ 20 mL/min/1.73 m^2^ and urine albumin–creatinine ratio ≥ 200 mg/g or heart failure, irrespective of albuminuria levels [15]. Across all SGLT2i indications, the treatment guidelines recommend use as a first-line option [14,15].

Previously, a decision analytical model was performed to quantify the number of deaths delayed or prevented with the addition of SGLT2i therapy to the management of HFrEF [7]. The model found that when utilized in appropriate patients, an SGLT2i can prevent around 34,000 deaths per year [7]. In addition to the analytical model, there have been several retrospective studies designed to assess the survival benefit associated with the utilization of GDMT for HFrEF [19]. One observational cohort study looked at 43,591 patients with heart failure and LVEF < 35% [19]. The investigators compared the mortality risk of different numbers of GDMT pillars with or without implantable cardioverter–defibrillators (ICDs) [19]. In the cohorts without an ICD, the estimated 24-month survival rates of 0, 1, 2, 3, and 4 GDMT classes were 63.7%, 66.5%, 72.0%, 76.9%, and 82.3%, respectively [19]. Based on these findings, with each additional GDMT class added to HFrEF management, the survival rate progressively increases, with the greatest benefit seen with all four pillars [19]. These two studies further strengthen the recommendation to optimize HFrEF GDMT and utilize as many classes as tolerated by the patient [7,19].

Despite the reduction in heart failure hospitalizations and mortality for HF with SGLT2i, in real-world utilization, several barriers have been discovered when initiating this medication class, including serious adverse effects. In 2015, the Food and Drug Administration (FDA) released a safety review and label revision to include a warning for ketoacidosis and serious urinary tract infections with the use of an SGLT2i [11]. This warning was published in response to 19 reported cases of hospitalization due to urosepsis and pyelonephritis that started as UTIs after SGLT2i use [11]. Since this announcement, clinical trials have reported the difference between placebo vs. SGLT2i in the incidence of UTIs [11]. When looking at the incidence of UTIs in clinical trials, for the DAPA-HF, EMPEROR-Reduced, SOLOIST-WHF, and DELIVER trials, there was no statistical difference in the number of UTIs [2,3,4,6]. The EMPEROR-Preserved trial was the only heart failure trial that showed an elevated risk of UTI with SGLT2i treatment (9.9% with SGLT2i vs. 8.1% with placebo) [5]. These trials and further real-world usage have shown that the concern for UTIs may not be as severe as initially thought [2,3,4,5,6,11].

Outside of clinical trials, a meta-analysis assessed the infection-related safety profile of SGLT2i in 86 smaller randomized controlled trials for a combined total of 50,800 included patients [13]. When comparing an SGLT2i to placebo (RR 1.03, 95% CI 0.96–1.11, I2 0%) or an active anti-diabetes comparator (RR 1.08, 95% CI 0.93–1.25, I2 22%), the SGLT2i did not show a significant difference in risk of UTI [13]. Dapagliflozin was the only SGLT2i that had a higher incidence of urinary tract infections compared to placebo (RR 1.23, 95% CI 1.03–1.46, I2 0%), and appeared to be a dose-dependent risk, with higher infection incidence with the 10 mg vs. 5 mg doses [13]. Regarding complicated UTIs, including urosepsis and pyelonephritis, there was a statistically higher number of infections associated with SGLT2i use; however, the wide range of results led to inconclusive findings [13]. Differing from previous studies, the investigators of this meta-analysis concluded that there is no significant association between SGLT2i use and UTI incidence, including complicated infections [13]. While this study is not completely applicable as it focused solely on patients with T2DM and does not include heart failure, the findings are still applicable due to diabetes being a risk factor for UTI [13].

The findings of our study could contribute to the existing literature on SGLT2i usage in the high-risk elderly population, given that our participants’ mean age was 66 years. In one European study, it was found that elderly patients with diabetes who were hospitalized for acute heart failure had statistically higher in-hospital mortality, 1-year all-cause mortality, and 1-year heart failure hospitalizations; even when adjusted for confounding variables, such as LVEF or kidney function [20]. This study is one of many that highlight the significance of diagnoses such as diabetes or heart failure on survival outcomes in this high-risk patient population [20]. Another study specifically focused on assessing the impact of comorbidities on hospitalized elderly patients with type 2 diabetes [21]. In older patients with diabetes, the authors found that increased heart rates and systolic blood pressure were associated with an increased risk of cardiovascular events/death and all-cause mortality [21]. The presence of comorbidities in elderly patients with diabetes appears to further increase mortality risk. This was further shown in a study where the authors graphically depicted comorbidities and their association with mortality via the “diabetes comorbidome” [22]. These authors found that in elderly patients with diabetes, having a co-diagnosis of heart failure statistically increased the risk of in-hospital, 3-month, and 1-year mortality [22]. When taken together, the findings of these studies highlight the importance of treating high-risk elderly patients with diabetes and heart failure due to their increased mortality risk. Utilization of GDMT first-line agents, such as SGLT2i, to treat heart failure could positively impact survival outcomes in these patients; therefore, adding evidence that there is not a higher risk of UTI despite history could help promote prescribing.

Our study included a total of 250 patients taking an SGLT2i, of whom only 53 (21.2%) had a history of UTIs. The incidence of post-SGLT2i UTIs was not found to be statistically significant when comparing UTI-naive (20.4%) and history of UTI (30.2%) cohorts (*p* = 0.13, Table 2). While the number of UTIs is noted to be higher than expected based on previous literature, this may have been due to the selection of eligible patients when pulling the initial data collection report. Based on our findings, having a history of UTI may not increase the risk of developing a UTI after starting an SGLT2i. This study supports that SGLT2is should not be withheld in patients solely based on the fear of recurrent UTIs in patients with a history of UTIs. Our subgroup analysis for patients with HF showed a significantly higher incidence of UTIs in patients without versus with a history of UTIs (68.2% vs. 31.8%, *p* = 0.04, Table 3). This suggests that all patients are at risk of developing a UTI with SGLT2i use in HF, regardless of the risk factor of a historical urinary infection. When specifically looking at HFrEF, there was only a 15.2% incidence of UTI out of the 52 patients, with no difference in the two cohorts (Table 3). In addition to the existing guidelines and trials proving the benefit of SGLT2i utilization in HF, this study can provide further confidence in prescribing to patients with historical UTIs. Previous notions surrounding the risk of SGLT2i causing urinary infections should still be noted in prescribing; however, the results of our study should help minimize this barrier in select patients.

There are several possible limitations to our study. First, since this was a small, retrospective study and only included 53 patients with a history of UTI prior to SGLT2i use, the applicability to real-world cases might differ. Additionally, only a small subset of patients, 52 out of 250, had HFrEF. Secondly, when compiling the list of possible patients to be analyzed for the study, this included patients taking an SGLT2i during their hospital admission and a diagnostic code for UTI and HF. Due to the initial data collection specifically looking for UTIs, this may have skewed our incidence numbers to reflect higher numbers of infections. Another limitation was the possibility of underrepresentation of the UTI incidence per patient. Since this was a single healthcare system review, the data may not have reflected every UTI developed if the patients went to other healthcare systems for treatment. Lastly, outside of the utilization of an SGLT2i, there may have been other confounding variables that led to urinary tract infections, which were not within the scope of this study to analyze. Despite its limitations, our study still adds to the literature surrounding SGLT2i-associated UTIs and provides some confidence in prescribing to patients with a historical UTI. The safety of initiating an SGLT2i in patients with urinary infections continues to be an area of discussion and would benefit from additional, larger studies to further add to the existing evidence.

## 5. Conclusions

While SGLT2i use may increase the number of UTIs a patient develops, having a history of recurrent UTIs should not be a barrier to withholding therapy in patients with HF. When comparing patients with and without a history of UTI prior to starting an SGLT2i, there was no statistical difference in post-SGLT2i UTI incidence. As a first-line recommendation in HFrEF and a reasonable option in HFpEF and HFmrEF, the benefits outweigh the risk of utilization of SGLT2i for heart failure management. These findings may help provide reassurance in prescribing an SGLT2i in patients with a history of UTIs, specifically in heart failure, where the benefits of GDMT have been previously proven.

## Figures and Tables

**Table 1:** Participant demographics and baseline characteristics.

Baseline Characteristics	All Patients (*N* = 250)	UTI-Naïve (*N* = 197)	History of UTI (*N* = 53)	*p*-Value
**Demographics**				
Age *, years	66 ± 13.1	67 ± 12.6	65 ± 15.1	0.33
BMI *, kg/m^2^	31.2 ± 7.4	30.9 ± 7.2	31.9 ± 8.4	0.39
Males, *n* (%)	114 (45.6%)	106 (53.8%)	45 (84.9%)	**<0.01**
**Race (*n*, %)**				
White	63 (25.2%)	51 (25.9%)	12 (22.6%)	0.72
Black	54 (21.6%)	48 (24.4%)	6 (11.3%)	0.04
Asian	6 (2.4%)	4 (2.0%)	2 (3.8%)	0.61
Other	125 (50.0%)	93 (47.2%)	32 (60.4%)	0.75
**Serum Levels**				
Serum Creatinine *, mg/dL	1.1 ± 0.45	1.1 ± 0.44	1.0 ± 0.49	0.15
Creatinine Clearance *, mL/min	88.9 ± 49.5	87.1 ± 42.5	95.7 ± 68.8	0.26
eGFR *, mL/min/1.73 m^2^	69.5 ± 28.9	69.5 ± 28.2	69.3 ± 31.4	0.96
**Comorbidities (*n*, %)**				
Heart Failure	108 (43.2%)	87 (44.2%)	21 (39.6%)	0.64
LVEF *, %	46.8 ± 14.2	47.5 ± 14.4	42.5 ± 17.7	**0.03**
HFrEF (LVEF < 40%)	52/108 (48.1%)	42/87 (48.3%)	10/21 (41.7%)	1.0
Type 2 Diabetes Mellitus	210 (84.0%)	160 (81.2%)	50 (94.3%)	**0.02**
**Prescribed SGLT2i (*n*, %)**				
Canagliflozin	1 (0.4%)	1 (0.5%)	0 (0.0%)	1.0
Dapagliflozin	48 (19.2%)	44 (22.3%)	4 (8.5%)	**0.02**
Empagliflozin	189 (75.6%)	142 (72.1%)	47 (88.7%)	**0.01**
Ertugliflozin	9 (3.6%)	8 (4.1%)	1 (1.9%)	0.69

* Mean ± standard deviation. BMI: body mass index; eGFR: estimated glomerular filtration rate; HFrEF: heart failure with reduced ejection fraction; LVEF: left-ventricular ejection fraction; SGLT2i: sodium-glucose cotransporter 2 inhibitors; UTI: urinary tract infection.

**Table 2:** Results of primary and secondary outcomes.

	All Patients (*N* = 250)	UTI-Naïve (*N* = 197)	History of UTI (*N* = 53)	*p*-Value
**Primary Outcome (n, %)**				
UTI incidence post-SGLT2i	56/250 (22.4%)	40/197 (20.4%)	16/53 (30.2%)	0.13
**Secondary Outcomes (n, %)**				
UTIs 0–30 days post-SGLT2i	6/66 (9.1%)	3/44 (6.8%)	3/21 (14.3%)	0.38
UTIs 30–60 days post-SGLT2i	2/66 (3.0%)	1/44 (2.3%)	2/21 (9.5%)	0.24
UTIs 60–90 days post-SGLT2i	8/66 (12.1%)	6/44 (13.6%)	2/21 (9.5%)	1.0
UTIs > 90 days post-SGLT2i	50/66 (75.8%)	34/44 (77.3%)	14/21 (66.7%)	0.38

**Table 3:** Subgroup analyses of UTI incidence.

	All Patients (*N* = 250)	UTI-Naïve (*N* = 197)	History of UTI (*N* = 53)	*p*-Value
**Subgroups (*n*, %)**				
Types 2 Diabetes, *n* = 210	53/210 (25.2%)	38/53 (71.7%)	15/53 (28.3%)	**<0.01**
Heart Failure, *n* = 108 *	22/108 (20.3%)	15/22 (68.2%)	7/22 (31.8%)	**0.04**
HFrEF, *n* = 52 **	8/52 (15.2%)	5/8 (62.5%)	3/8 (37.5%)	0.62
HFmrEF, *n* = 20	6/20 (30.0%)	4/6 (66.7%)	2/6 (33.3%)	0.59
CKD, *n* = 77	19/77 (24.7%)	10/19 (52.7%)	9/19 (47.4%)	1.0
**Prescribed SGLT2i (*n*, %)**				
Empagliflozin, *n* = 189	46/189 (24.3%)	34/46 (73.9%)	12/46 (26.1%)	**<0.01**
*5 mg*, *n* = *2*	1/2 (50.0%)	1/1 (100%)	0/1 (100%)	1.0
*10 mg*, *n* = *142*	32/142 (22.5%)	8/32 (25.0%)	24/32 (75.0%)	**<0.01**
*25 mg*, *n* = *45*	13/45 (28.9%)	9/13 (69.2%)	4/13 (30.8%)	0.12
Dapagliflozin, *n* = 48	6/48 (12.5%)	3/6 (50.0%)	3/6 (50.0%)	1.0
*5 mg*, *n* = *2*	1/2 (50.0%)	1/1 (100%)	0/1 (0%)	1.0
*10 mg*, *n* = *46*	5/46 (10.9%)	2/5 (40.0%)	3/5 (60.0%)	1.0
Canagliflozin, *n* = 1	1/1 (100%)	1/1 (100%)	0/1 (0%)	1.0
Ertugliflozin, *n* = 9	2/9 (22.2%)	2/2 (100%)	0/1 (0%)	1.0

CKD: chronic kidney disease (defined as eGFR < 60); HFmrEF: heart failure with moderately reduced ejection fraction; HFrEF: heart failure with reduced ejection fraction. * Most HF participants also had T2DM comorbidity; only 2 participants did not have T2DM. ** Most HFrEF participants also had T2DM comorbidity; only 1 participant did not have T2DM.

**Table 4:** Comparison of Pathogens pre-SGLT2i and post-SGLT2i use.

Pathogens	Pre-SGLT2i (*n*,%)	Post-SGLT2i (*n*, %)
*Acinetobacter ursingii*	1 (1.6%)	0 (0%)
*Aerococcus* spp.	1 (1.6%)	1 (1.0%)
*Candida* spp.	3 (4.7%)	8 (8.0%)
*Citrobacter* spp.	3 (4.7%)	1 (1.0%)
*Escherichia coli*	30 (46.9%)	38 (38.0%)
*Escherichia coli ESBL*	5 (7.8%)	8 (8.0%)
*Enterococcus faecalis*	1 (1.6%)	10 (10.0%)
*Enterobacter* spp.	0 (0%)	2 (2.0%)
*Group B Streptococcus*	5 (7.8%)	7 (7.0%)
*Klebsiella pneumoniae*	9 (16.7%)	17 (17.0%)
*Lactobacillus*	0 (0%)	1 (1.0%)
*MRSA*	1 (1.6%)	0 (0%)
*MSSA*	2 (3.1%)	2 (2.0%)
*Proteus* spp.	2 (3.1%)	0 (0%)
*Pseudomonas*	1(1.6%)	3 (3.0%)
*Salmonella*	0 (0%)	1 (1.0%)
*Staphylococcus simiae*	0 (0%)	1 (1.0%)
**Total UTI Pathogens (n)**	64	100

ESBL: extended-spectrum beta-lactamase; MSSA: methicillin-susceptible *Staphylococcus aureus*; MRSA: methicillin-resistant *Staphylococcus aureus*; spp.: species.

## Data Availability

The raw data supporting the conclusions of this article will be made available by the authors upon request.

## Supplementary Materials

The following supporting information can be downloaded at: https://www.mdpi.com/article/10.3390/healthcare14030318/s1, Table S1: Comparison of UTI pathogens in participants with pre-SGLT2i and post-SGLT2i UTIs.

## Abbreviations

The following abbreviations are used in this manuscript:

ACC	American College of Cardiology
AHA	American Heart Association
ASCVD	Atherosclerotic cardiovascular disease
BMI	Body mass index
CI	Confidence interval
CKD	Chronic kidney disease
DPP4i	Dipeptidyl peptidase-4 inhibitors
eGFR	Estimated glomerular filtration rate
FDA	Food & Drug Administration
GDMT	Guideline-directed medical therapy
GLP-1 RA	Glucagon-like peptide 1 receptor agonist
HF	Heart failure
HFmrEF	Heart failure with moderately reduced ejection fraction
HFpEF	Heart failure with preserved ejection fraction
HFrEF	Heart failure with reduced ejection fraction
HR	Hazard ratio
ICD-10	International Classification of Diseases 10
ICDs	Implantable cardioverter–defibrillator
KDIGO	Kidney Disease Improving Global Outcomes
LVEF	Left ventricular ejection fraction
MACE	Major adverse cardiovascular events
SD	Standard deviation
SGLT2i	Sodium-glucose co-transporter-2 inhibitors
T2DM	Type 2 diabetes mellitus
UTI	Urinary tract infection
