# Supplementary material for: Safety of Initiating Sodium-Glucose Cotransporter-2 Inhibitors in Patients with Heart Failure or Type 2 Diabetes and a History of Urinary Tract Infections
Source: Healthcare (Basel). 2026 Jan 27;14(3):318. doi: 10.3390/healthcare14030318 (PMC12897258; doi:10.3390/healthcare14030318)
Supplement: Supplementary file 1 [file healthcare-14-00318-s001.zip › healthcare-4009108-supplementary.pdf]

	Prior UTI (pre-SGLT2i)				UTI post-SGLT2i		
**Participant ID**	**Number**	**Time Since Last UTI**	**Complicated (Y/N)**	**Pathogen(s)**	**Time after SGLT2i**	**Complicated (Y/N)**	**Pathogen(s)**
10	2	5 months	N	Candida albicans<br>MSSA	> 1 year	N	Lactobacillus<br>Candida glabrata<br>Staph simulans
24	2	1 months	N	E. coli<br>Kleb pneumo	6 months	N	E. coli
29	2	> 1 year	N	Kleb pneumo<br>Acinetobacter ursingii	3 months	N	E. coli<br>Group B Strep<br>MSSA<br>Candida albicans
51	1	5 months	Y	E. faecalis	6 months	Y	E. faecalis
62	1	> 1 year	N	Citrobacter	5 months	N	E. faecalis
147	1	2 months	Y	MRSA	< 30 days	N	Kleb pneumo
220	5	0 days	N	E. coli<br>Kleb pneumo x 4	< 30 days	N	Kleb pneumo
236	1	> 1 year	N	E. coli	> 1 year	N	Pseudomonas<br>E. faecalis<br>Group B Strep
239	2	14 days	N	Citrobacter<br>E. coli	> 1 year	N	E. coli
328	1	4 months	N	MSSA	9 months	Y	Kleb pneumo
359	1	> 1 year	N	E. coli	9 months	N	E. coli
371	1	7 days	N	GNR	< 30 days	N	Enterobacter
404	1	> 1 year	N	E. coli ESBL	> 1 year	N	E. coli
458	1	2 months	N	Group B Strep	> 1 year	N	E. faecalis
479	1	5 months	N	E. coli	> 1 year	N	E. coli
485	2	4 months	N	E. coli<br>Aerococcus	> 1 year	N	Pseudomonas

Supplemental Table S1: Comparison of UTI pathogens in participants with pre-SGLT2i and post-SGLT2i UTIs
